# Molecular prevalence of *Toxoplasma gondii* DNA in goats’ milk and seroprevalence in Northwest Tunisia

**DOI:** 10.1002/vms3.29

**Published:** 2016-03-07

**Authors:** Safa Amairia, Mariem Rouatbi, Mohamed R. Rjeibi, Hanen Nouasri, Limam Sassi, Moez Mhadhbi, Mohamed Gharbi

**Affiliations:** ^1^ Laboratoire de Parasitologie Univ. Manouba Institution de la Recherche et de l'Enseignement Supérieur Agricoles École Nationale de Médecine Vétérinaire de Sidi Thabet Sidi Thabet 2020 Tunisia

**Keywords:** *Toxoplasma gondii*, goat, milk, ELISA, PCR, Tunisia

## Abstract

Toxoplasmosis is a worldwide zoonosis with high impact on human and animal health. Consumption of unpasteurized milk is a risk factor of human toxoplasmosis. The aim of this study was to estimate the seroprevalence and molecular prevalence of *T. gondii* in goats’ milk in Northwest of Tunisia (Jendouba Governorate). A total number of 77 blood samples were collected from six herds were screened with a commercial ELISA kit for *T. gondii* antibodies. For the same goats’ samples, a nested PCR was performed to detect *T. gondii* DNA in milk. The seroprevalence of *T. gondii* infection was 31.2% (±0.05) while the molecular prevalence of this parasite in milk was estimated to 7.8% (±0.03). A very low value of kappa showed that there is not agreement between seroprevalence and parasite prevalence in milk. These results suggest that the consumption of raw milk from naturally infected goats is a potential source of human infection. An extension programme should be implemented to decrease related to goats’ raw milk consumption.

## Introduction

Toxoplasmosis is a zoonotic infection caused by *Toxoplasma gondii*, a protozoan parasite belonging to the order Coccidia and the Apicomplexa phylum. *T. gondii* infects warm‐blooded animals including humans with felines (mainly cats) as definitive hosts (Dubey 2010). Toxoplasmosis is a serious risk for seronegative pregnant women and immunocompromised persons (Montoya & Liesenfeld 2004; Tenter 2009). Infection with *T. gondii* is frequently asymptomatic, but remains serious even fatal for specific groups including congenitally infected fetuses and newborns, immunocompromised individuals (AIDS patients), and transplanted persons (Saadatnia & Golkar 2012). It is estimated that *T. gondii* infects up to one‐third of the human population in the world (Dubey & Jones 2008). In Tunisia, human seroprevalence is high; it has been estimated to 70% in female Tunisian aged of 30 years in the northwest of the country (Bouratbine *et al*. 2001).

Among the food‐borne diseases, toxoplasmosis is a real burden in several countries (EFSA, 2007; Havelaar *et al*. 2010; Scallan *et al*. 2011). For example, in USA, toxoplasmosis is the second cause of mortality after salmonellosis, and the fourth cause of hospitalization after salmonellosis, campylobacteriosis and norovirus infections (Scallan *et al*. 2011). Many foods are a reservoir of *T. gondii*. The main concerned foods are raw and undercooked meat infected with *T. gondii,* unpasteurized milk from infected animals, infected vegetables and water contaminated with oocysts (Dubey 1994, 1996; Baril *et al*. 1995). Among food animals, sheep and goats are the main sources of human infection (Dubey 2010). Infection with *T. gondii* in goats is ranging between 3.7 and 81.8% (Dubey & Adams 1990; Costa *et al*. 2012). In North Africa, studies about *T. gondii* infection in goats are scarce. In Egypt, positive ELISA and PCR results were found in 41.7 and 25% of goats, respectively (Ghoneim *et al*. 2010). In Tunisia, a single study was performed by Ben (1986), the seroprevalence in goats was estimated to 16%. Recently, a serological survey carried out in Morocco, reported that 8.5% (9/106) of goats were positive to toxoplasmosis (Benkirane *et al*. 2015). Several studies showed that drinking unpasteurized goats’ milk could cause clinical and even fatal toxoplasmosis infections in humans (Riemann *et al*. 1975; Patton *et al*. 1990; Skinner *et al*. 1990). When excreted in milk, *T. gondii* tachyzoites are infective (Skinner *et al*. 1990). Recently, few studies indicated the presence of *T. gondii* in goat's milk and reported low prevalence ranging between 6 and 9.4% (Bezerra *et al*. 2013; Dehkordi *et al*. 2013).

Among the reliable methods, polymerase chain reaction (PCR) has the highest accuracy, sensitivity and specificity compared with conventional diagnostic methods (Held *et al*. 2000; Kompalic‐Cristo *et al*. 2007). This study aimed to estimate the seroprevalence of *T. gondii* infection and the molecular prevalence of *T. gondii* DNA in milk samples to provide a preliminary assess of contamination risk by *T. gondii* in Tunisian goats’ milk.

## Materials and methods

### Study area

**Figure 1:**
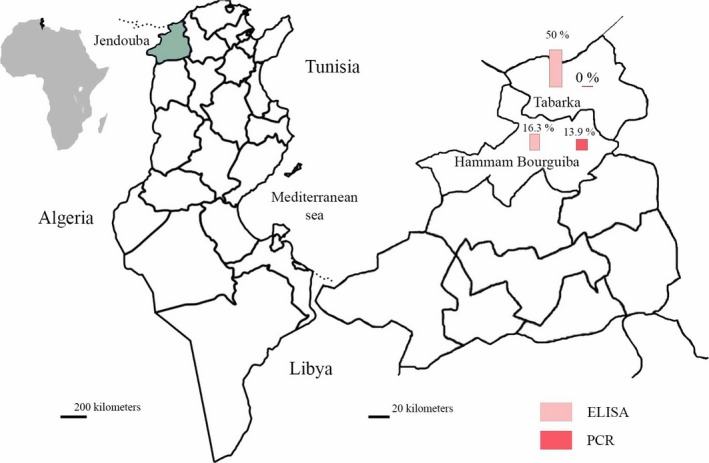
Goat ELISA and PCR infection prevalence of *Toxoplasma gondii* in the governorate of Jendouba (districts of Hammam Bourguiba and Tabarka, Northwest Tunisia)

This study was carried out in goat farms located in Northwest Tunisia (localities of Tabarka and Hammam Bourguiba, Jendouba Governorate (Fig. 1)). These localities are situated between 36°45′ and 36°57′ North latitudes and between 8°35′ and 8°45′ East longitudes with an altitude ranging between 20 and 150 m. The governorate of Jendouba has the most temperate climate in Tunisia with a mean annual rainfall of 1000 mm. The mean minimal and maximal temperature are 5 and 30°C in winter and summer, respectively (National Institute of Meteorology, Tunisia).

### Samples collection

This study was carried out from February to May 2014 in six herds totalling 77 lactating goats of different ages and breeds (Table 1). Blood samples were collected in dry tubes from the jugular vein; the sera were separated and stored at −20°C until used. Simultaneously, 50 mL of milk was collected from each goat and kept frozen in sterile tubes for molecular study.

**Table 1:** Association between *Toxoplasma gondii* prevalence in goats and different parameters

Parameter		ELISA		PCR	
		Positive/examined (%±SE)	OR [95% CI]	Positive/examined (%±SE)	OR [95% CI]
Locality	Tabarka Hammam Bourguiba	17/34 (50 ± 9) 7/43 (16.3 ± 6)	5.14 [1.61; 16.99]*	0/34 (0)* 6/43 (13.9 ± 5)	NA
Age group (years)	≤3 4 to 5 ≥6	9/19 (47.4 ± 11) 9/42 (21.4 ± 6) 6/16 (37.5 ± 12)	3.3 [0.89; 12.49] 1.5 [0.32; 7.26]	2/19 (10.5 ± 7) 3/42 (7.1 ± 4) 1/16 (6.2 ± 6)	1.53 [0.16; 12.89] 1.76 [0.11; 54.96]
Breed	Local breed Cross bred Exotic breeds*	5/22 (22.7 ± 9) 10/31 (32.2 ± 8) 9/24 (37.5 ± 9)	0.62 [0.15; 2.51] 0.49 [0.11; 2.12]	2/22 (9.1 ± 6) 4/31 (12.9 ± 6) 0/24	0.68 [0.08; 4.99] NA
Abortion history	Yes No	5/19 (26.3 ± 10) 19/58 (32.7 ± 6)	0.73 [0.2; 2.64]	4/19 (21 ± 9) 2/58 (3.4 ± 2)	7.47 [1.02; 66.13]*
Number of cats	0 1 ≥2	6/8 8/49 (16.3 ± 5) 10/20 (50 ± 11)	15.38 [2.16; 137.78]* 3 [0.38; 28.43]	0/8 6/49 (12.2 ± 5) 0/20	NA

SE, Standard Error; CI, 95% Confidence Interval; NA, Not Applicable. *P ≤ 0.05. *Alpine and Boer goats.

### Serology

Samples of 10 *μ*L of sera were examined for IgG antibodies against *T. gondii* using an ELISA kit (ID Screen^®^ Toxoplasmosis Indirect, Montpellier, France) in accordance to the manufacturer's instructions.

### Molecular study

DNA was extracted from milk as described by Mancianti *et al*. (2013). Briefly, the milk samples were centrifuged at 2200 *g* for 5 min. To avoid interference with casein, 1 mL of the pellet was treated with 200 *μ*L of TE (1 mM EDTA, 10 mM Tris‐HCl (pH = 7.6)) and 300 *μ*L 0.5 M EDTA (pH 8). The solution was resuspended then centrifuged at 3000 *g* for 10 min and finally diluted in 200 *μ*L of PBS (Psifidi *et al*. 2010). DNA was isolated from the resuspended pellet using Wizard Genomics DNA (Promega, Madisson, Wisconsin, USA) extraction kit according to manufacturer's instructions; the DNA was stored at −20°C until used.

To evaluate the efficiency of DNA extraction protocol, a PCR assay targeting the hypervariable regions V1‐V3 coding for 18S rRNA was performed using two forward primers AACCTGGTTGATCCTGCCAGT (1 A) and reverse primers GGCACCAGACTTGCCCTC (564 R). The PCR reaction was carried out in a mixture of 1× PCR buffer, 2 mM MgCl_2_, 10 *μ*M of each primer, 0.2 mM of each dNTP, 2 U Taq polymerase, 1.5 *μ*L of DNA template and distilled water to a total volume of 25 *μ*L (Wang *et al*., 2014). The PCR conditions were 5 min at 94°C followed by 25 cycles of denaturation at 94°C for 50 s, annealing at 58°C for 50 s and a final extension at 72°C for 10 min. A nested PCR was performed to amplify a *T. gondii* DNA fragment of 227 bp belonging to ITS1 gene and coding for the 18S – 5.8S rRNA according to a modified protocol of Hurtado *et al*. (2001). Two external primers were used, namely, NN1 (5′‐CCTTTGAATCCCAAGCAAAACATGAG‐3′) and NN2 (5′‐GCGAGCCAAGACATCCATTGCTGA‐3′). After the first PCR, nested PCR was performed using 2 *μ*L of the first PCR product as template and *T. gondii*‐specific primers Tg‐NP1 (5′‐GTGATAGTATCGAAAGGTAT‐3′) and Tg‐NP2 (5′‐ACTCTCTCTCAAATGTTCCT‐3′). Each amplification was performed in 25 *μ*L of total reaction volume consisting of 0.1 *μ*M of each primer, Taq polymerase buffer (1x) supplemented with MgCl_2_ (2 mM), 0.2 mM dNTPs, 0.5 U Taq DNA polymerase (R GoTaq, Promega). Thermal amplification programme was 3 min at 94°C followed by 30 cycles of denaturation at 94°C for 30 s, annealing at 67°C for 45 s and extension at 72°C for 1 min with an elongation step of 5 min at 72°C. For nested PCR, all thermal cycler conditions were the same, except the annealing temperature (53°C for 30 s). Positive controls (including *T. gondii* DNA) and negative controls (distilled water) were included in each PCR run. The PCR products were visualized by electrophoresis in 1.5% (w/v) agarose gel (Promega^®^) supplemented with 0.05% ethidium bromide (Promega^®^) in TBE buffer (Tris‐Borate‐EDTA) (Promega^®^).

### Statistical analysis

A chi‐square Mantel–Haenszel test was performed for comparison of infection prevalence with Epi Info 6 at a threshold of 5% (Schwartz 1993). The concordance between PCR and ELISA was estimated with the Kappa test (Toma *et al*. 2007).

## Results

### Seroprevalence

The seroprevalence in goats was 31.2% (±5%). It was significantly higher in Tabarka (17/34; 50 ± 9%) than in Hammam Bourguiba (7/43; 16.3 ± 6%) (*P* = 0.001) (Table 1). There was no significant difference in the seroprevalence of *T. gondii* in different age categories. The infection rate by *T. gondii* was higher in herds containing more than one cat (*P* = 0.004).

### Molecular detection in milk

A total number of six samples milk were positive (7.8 ± 3%). The *T. gondii* molecular prevalence was significantly higher in Hammam Bourguiba (6/43; 13.9 ± 5%) than in Tabarka (0/34) (*P* = 0.02). The molecular prevalence of *T. gondii* was higher for goats with history of abortion (21 ± 9.4%) (*P* = 0.01). Among seropositive goats (*n* = 24), two milk samples were positive to n‐PCR (8.33 ± 5%). The kappa coefficient between n‐PCR and ELISA was very low (Ƙ = 0.01).

### Discussion and conclusion

In this study, we used an ELISA and a nPCR in milk for detection of *T. gondii* from lactating goats in North‐western Tunisia. Anti‐*T. gondii* antibodies were found in 31.2% of serum samples, only two of them were also positive for milk‐PCR. Our results were comparable with those reported in China (29.54% by ELISA), Thailand and Pakistan (27.9 and 25.5% by Latex Agglutination Test, respectively) (Jittapalapong *et al*. 2005; Ramzan *et al*. 2009; Liu *et al*. 2015). In Europe, *T. gondii* is widespread with high infection rates. In Italy, 60.6% of goats’ serum samples were positive by micro‐agglutination test (Mancianti *et al*. 2013). Similar findings were reported in Bulgaria, where 59.8% of the samples were positive by inhibition of haemagglutination test (Prelozov *et al*. 2008). In Romania, the seroprevalences were 33.1 and 52.8% in kids and dairy goats, respectively (Lovu *et al*. 2012; Paştiu *et al*. 2015). In USA, Korea and Nigeria lower rates were reported (6.8; 5.1 and 4.6%, respectively) (Kamani *et al*. 2010; Jung *et al*. 2014; Yaglom *et al*. 2014).

Consuming raw goats’ milk represents a real risk factor for human toxoplasmosis (Skinner *et al*. 1990). Detection of toxoplasmosis in different lactating species was recently studied by Dehkordi *et al*. (2013), with different techniques. They showed that raw milk from all domestic species (goats, sheep, buffalos, cattle and camel) could be contaminated. The prevalence of *T. gondii* in the milk of naturally infected goats is relatively low. We found that 7.8% of milk samples were shedding *Toxoplasma* DNA. Similar results were reported by Mancianti *et al*. (2013) in Italy where 7.9% of the samples were positive. A slightly lower rate was reported in Brazil where the parasite was detected in 6% of the milk samples (15/248) (Bezerra *et al*. 2013). The environmental conditions, diagnostic methods and the presence of cats in the herds could explain this discrepancy. In this study, the molecular prevalence of *T. gondii* was significantly higher in Hammam Bourguiba than in Tabarka (*P* = 0.02). This might be attributed to its climate which is characterized by a high rainfall enhancing oocysts dispersion.

Our results showed also that the infection rate was higher for goats with abortion history (*P* = 0.01). Since congenital toxoplasmosis is one of the main causes of abortion in goats (Duncanson *et al*. 2001), the consumption of raw milk from infected goats may present a real risk to public health.

There was no concordance between the two techniques (4/77; 5.2%). Positive PCR and negative ELISA samples could be due to the occurrence of early infections (Bezerra *et al*. 2013). Further studies are needed to quantify and estimate the viability of *T. gondii* excreted in the milk. Our results provided a molecular evidence of *T. gondii* DNA presence in raw goats’ milk. Considering these results, authorities must integrate health education guidance to encourage milk consumers to avoid drinking raw milk. Toxoplasmosis infection must therefore be added to the list of pathogens transmitted by raw milk with *Brucella melitensis* (Gupta *et al*. 2006).

## Source of funding

The work was funded by the laboratory of “Laboratoire d’épidémiologie des infections enzootiques des herbivores en Tunisie: application à la lutte” (Ministère de l'enseignement supérieur et de la recherche scientifique, Tunisia).

## Conflicts of interest

The authors declare no conflict of interests in relation to this work.

## Contributions

SA, MG, MRR and MM conceived and designed the experiments. SA and MR performed the experiments. HN involved in the collection of samples. LS contributed to the analysis of tools. SA and MG wrote the manuscript.
